# Metabolic Shifts Induced by Treatment with Statin Influences Circulating Concentrations of the Stress Hormone, Cortisol, but Has Different Effects on Selected Cytokines, Adipokines and Neuropeptides in Lean and Fat Lines of Young Pigs

**DOI:** 10.3390/metabo15120797

**Published:** 2025-12-16

**Authors:** Krystyna Pierzchała-Koziec, Colin G. Scanes, Joanna Zubel-Łojek, Mirosław Kucharski

**Affiliations:** 1 Department of Animal Physiology and Endocrinology, University of Agriculture, Mickiewicza 24/28, 30-059 Kraków, Poland; joanna.zubel@urk.edu.pl (J.Z.-Ł.); miroslaw.kucharski@urk.edu.pl (M.K.); 2 Department of Biological Science, University of Wisconsin Milwaukee, Milwaukee, WI 53211, USA; scanes@uwm.edu

**Keywords:** stress, statin, cytokines, adipokines, epicardial fat, visceral fat, piglets

## Abstract

**Background**: It was hypothesized that short statin treatment would shift adipose expression, plasma and adipose tissue concentrations of adipokines and cytokines. **Methods**: Effects of a statin administration on adipokines (leptin, resistin and visfatin), adipose inflammatory cytokines [interleukin-6 (IL-6) and tumor necrosis factorα (TNFα)] were examined in young pigs of both lean and fat breeds. **Results**: Expression of resistin was increased while that of visfatin was decreased in visceral adipose tissue and, to a lesser extent, in epicardial adipose tissue. In young pigs treated with statin, there were increases in the plasma concentrations of leptin, resistin and TNFα. There were also decreases in the plasma concentrations of visfatin, cortisol, Met-enkephalin and endothelin-1. Concentrations of leptin in both epicardial and visceral adipose tissue were reduced in statin-treated pigs. There were marked differences between the epicardial and visceral adipose tissue. Concentrations of leptin were reduced with statin treatment in visceral adipose tissue irrespective of whether they were lean or fat breeds of pigs. Statin treatment was associated with increased concentrations of TNFα in epicardial adipose tissue and of IL-6 in visceral adipose tissue in both lean and fat breeds of pigs. **Conclusions**: It is concluded that statins cause shifts in the expression and/or concentrations of both adipokines and inflammatory cytokines in adipose tissue.

## 1. Introduction

The present study examined the effects of the statin, atorvastatin (HMG-CoA reductase inhibitor), on plasma concentrations of cortisol, plasma, visceral and epicardial adipose tissue concentrations of adipokines, namely, ghrelin, leptin, resistin, visfatin and the pro-inflammatory cytokines tumor necrosis factor α (TNF-α) and interleukin-6 (IL-6) together with expression of two adipokines, resistin and visfatin. The study employed young pigs from both a lean and a fat breed, the latter as a model for obesity.

The overall question was to determine whether the metabolic stress induced by statin administration can influence the following: 1. adipocytes functioning based on synthesis, storage and release of adipokines; 2. release of pro-inflammatory cytokines from macrophage and other immune cells present in adipose tissue; 3. compare the parameters changes in lean and fat piglets. Macrophages represent not only greater than over half the interstitial cells in adipose tissue but also interact with adipocytes (reviewed: [1]). Adipose pro-inflammatory macrophages release cytokines, including TNF-α and IL-6, and these play a role in regulating adipocyte functioning (reviewed: [2]). In addition, IL-6 is also expressed by pre-adipocytes while TNF-α is expressed by adipocytes [3]. There are effects of adipose macrophages on adipocytes, and it was found that differentiation of pre-adipocytes is depressed in the presence of TNF-α [4]. Expression of resistin by adipocytes is influenced by IL-6 [4]. Leptin is produced by adipocytes [5,6], and this not only acts in an endocrine but also in a paracrine manner influencing immune cells [7]. Furthermore, another hormone stimulating feeding, ghrelin, influences differentiation of pre-adipocytes [8]. In spite of much research on the effects of statins administration (lipid metabolism) and their side effects, there is scarce data about the pleiotropic impact of statin on hormones and neuropeptides regulation. Also, the question arises how genetic background modulates the sensitivity to statins.

Therefore, it was hypothesized that the effects of statin would be the same directionality in the two breeds but may vary in magnitude.

## 2. Material and Methods

All animal procedures were conducted with prior institutional ethical approval in accordance with the Local Institutional Animal Care and Use Committee (IACUC). The animal study was approved by the Institutional Review Board and the First Local Ethical Committee on Animal Testing in Krakow, Poland (77/2008 and 120/2013). The experiment was conducted in accordance with the principles and specific guidelines presented in the Guide for the Care and Use of Agricultural Animals in Research and Teaching, 4th edition, 2020.

**Animals** The study employed 6-week-old female piglets (body weight 10–12 kg) of two breeds: 1. Polish Landrace (PBZ)—a fast-growing low-fat breed (Polish Land Race or PBZ termed lean) and 2. Puławska—a high-fat slow-growing breed (termed fat). The Puławska breed was developed in 1920s at an institute in the town Puławy in the village of Gołąb from local wild-type breeds and Berkshire pigs [9,10]; (reviewed: [11]). Piglets were purchased from the National Research Institute of Animal Production in Balice, Poland. Animals were housed in individual environmentally controlled pens maintained at 20–22 °C with a 12:12 h light–dark cycle. Animals were fed a commercial feed with a standard grain-based diet fulfilling their daily maintenance requirements and had free access to water for one week before the experiment.

**Experimentation** Young pigs were randomly assigned to two treatment groups: 1. control: (sham, received orally placebo); 2. statin (atorvastatin) administered orally at a dose of 20 mg/piglet, daily, for 5 consecutive days (randomly selected, double-blinded). There were five replicates (n = 5) per treatment group for each of the two breeds of pigs. Piglets received feed 2 h after the statin treatment.

The dose of statin was calculated as 1.7 to 2.0 mg/kg b.w. given at 8.00 a.m. The calculation was taking into account: 12% of bioavailability, Cmax 1–2 h, 24 s of blood circulation and an approximate 12–14 h of half-life (T1/2).

Statin was administered only for 5 days in order to test the early responses of parameters in healthy, but with different genetic background piglets. The pilot study showed that longer treatment could induce the process of adaptation in many tissues and the early responses to statin might be concealed [12,13,14,15].

**Blood samples** were taken from the external jugular vein 24 h after the last statin administration (8.00 a.m. on day six) and after centrifugation (30 min at 4 °C and 4000× *g*), plasma was stored at −80 °C prior to the assay of cholesterol, glucose, hormones, adipokines and cytokines. Hypothalamus, pituitary and adrenal explants were taken and directed to in vitro short tissues culture or frozen for determination of expression of visfatin and resistin. Samples of visceral and epicardial adipose tissues were taken for the determination of adipokines concentrations, pro-inflammatory cytokines and determination of the expression of resistin and visfatin.

### 2.1. Tissue Culture

Explants of the hypothalamus, pituitary gland and adrenal gland (each fragment 50–70 mg) were dissected and were placed on a 24-well plate. Tissues were incubated in 1 mL of Eagle’s medium supplemented with 0.05% bovine serum albumin and 2 μL of antibiotic–antimycotic solution (n = 5) for 60 min (30 min of basal and 30 min of stimulation) at 38 °C (5% CO_2_) in the Eagle’s medium in the presence (100 nM of naltrexone) or absence (basal release). Following incubation, the culture media were stored at −80 °C for ghrelin determination.

### 2.2. Assays

**Glucose and Cholesterol:** Plasma concentrations of glucose and cholesterol were determined using commercial colorimetric kits (AlphaDiagnostic, Warsaw, Poland).

**Insulin:** Plasma concentrations of insulin were determined using a commercial kit (Porcine Insulin RIA, Millipore, Burlington, MA, USA). The assay sensitivity was 1.6 μIU/mL and intra- and interassay coefficients of variance (CV) were 5% and 10%, respectively.

**Cortisol:** Plasma concentrations of cortisol were measured by radioimmunoassay employing commercial kits from DiaSorce (Mont-Saint-Guibert, Belgium). The interassay and intraassay were 6% and 12%, respectively.

**Met-enkephalin**: Concentrations of **Met-enkephalin** in the plasma were determined by radioimmunoassay [16]. The interassay and intraassay coefficients of variance were, respectively, 7% and 11%.

**Ghrelin:** Total ghrelin immunoreactivity was measured using RIA (Linco Research Inc., Minneapolis, MN, USA) with a sensitivity of 93 pg/mL and an interassay CV of 14.7% at 1000 pg/mL and 16.7% at 3000 pg/mL.

**Leptin:** was estimated by Multi-Species Leptin RIA Kit (Millipore, Burlington, MA, USA) with an intraassay CV of 3.2%, interassay CV of 7.8% and lowest measurable concentration was 0.2 pg/mL.

**Endothelin:** was measured using Endothelin 1 (EDN1) ELISA Kit (MyBiosource INC. San Diego, CA, USA) with the lowest detectable concentration of 0.5 pg/mL.

**Il-6** was determined using ELISA kit manufactured by RayBiotech (Peachtree Corners, GA, USA) with the lowest detectible concentration being 1.3 pg/mL and an intraassay CV < 10% and interassay CV < 12%.

**TNFα:** TNFα was determined by Porcine TNF-alpha ELISA Kit, (ELP-TNFa, RayBiotech, Peachtree Corners, GA, USA) with the lowest detectible concentration being 14 pg/mL, and the CV for intraassay being <10% and CV for interassay being <12%.

**Resistin:** Resistin was measured by standard commercial ELISAs according to the manufacturers’ recommended protocols (BioVendor, Brno, Czech Republic). The intra and interassay CV for the resistin ELISA were 5.9% and 7.6%, respectively. The assay can measure a concentration as low as 0.012 ng/mL and is sensitive and specific enough to measure resistin protein in various adipose tissues.

**Visfatin:** Visfatin was measured by commercial ELISA (BioVendor, Brno, Czech Republic). The intra- and interassay CV were 6.6% and 8.7%, respectively. The calibration range was from 0.063 to 16 ng/mL, and the limit of detection was 20 pg/mL.

### 2.3. RNA Extraction and cDNA Synthesis

Total RNA was extracted from adipose tissues using the TRIzol reagent (Leica Biosystems, Deer Park, IL, USA) according to the modified Chomczynski and Sachci method [17]. The quality and concentration of the obtained total RNA were checked by the NanoDrop (Thermo Fisher Scientific, Mount Prospect, IL, USA). High-quality RNA samples were then reversely transcribed into first strand cDNA immediately using the High Capacity RNA-to-cDNA Kit (Thermo Fisher Scientific, Mount Prospect, IL, USA). cDNA samples were stored in −80 °C and then used for RT-PCR analysis.

### 2.4. Real-Time PCR (qPCR)

The primers were designed based on the porcine genes sequences –resistin, visfatin and 18S rRNA as a housekeeping gene (Table 1).

The qPCR assays were performed using the StepOne Plus (Applied Biosystems, Carlsbad, CA, USA) on a 96-well. Detection of each sample was performed simultaneously 3 times. The procedure for qPCR was as follows: predenaturation at 95 °C for 15 min, followed by 45 cycles of 95 °C for 15 s, and 62 °C for 20 s and 72 °C for 20 s. qPCR reaction was performed using PowerUp™ SYBR™ Green Master Mix (Thermo Fisher Scientific, Mount Prospect, IL, USA).

Gene expression levels were normalized to the geometric mean of two reference genes, GAPDH (glyceraldehyde-3-phosphate dehydrogenase) and 18S rRNA (18S ribosomal RNA) using geNorm. The default threshold for acceptable stability has been set to a value of M below 1.5. Unfortunately, due to very high Ct values (approx. 30) and M > 1.5, the GAPDH gene was removed from further calculations. The primers were designed based on the sequence from the NCBI database. The reaction efficiency for each gene was determined before the experiment. The reaction efficiency values for all genes were within the range of 85–100%. The individual gene expression level was calculated by relative quantitative analysis and the Pfaffl model (the modification of 2^−ΔΔCt^ method), including the reaction efficiency for individual genes (StepOne Software v2.1. Applied Biosystems) [18].

**Statistics:** Data were analyzed by two-way ANOVA for breed and statin treatments. Where main effects or interaction to be significant (*p* < 0.05), means were separated using Tukey’s Honest Significant Difference (HSD) as the range test. Data on the two-way ANOVA are provided in Supplementary Tables (Tables S1–S8).

## 3. Results

### 3.1. Plasma Concentrations of Cholesterol, Glucose, Insulin and Cortisol

There were effects of statin administration, breed and interactions between these (*p* < 0.001) on plasma concentrations of cholesterol, glucose and insulin (Table 2). Plasma concentrations of cholesterol were decreased (*p* < 0.001) following statin administration in young pigs of the fat breed but not lean breed (Table 2). The former is what would be expected, but the absence of an effect of statin in the lean breed was not. In contrast, plasma concentrations of glucose were elevated (*p* < 0.001) in young pigs of the lean breed but were not affected in the fat breed. Statin administration was accompanied by decreases (*p* < 0.001) in plasma concentrations of insulin in fat breed young pigs. There were effects of statin administration and breed (*p* < 0.001) but no interactions between these on plasma concentrations of cortisol (Table 2). Statin administration was accompanied by decreased (*p* < 0.001) plasma concentrations of cortisol in both lean and fat breed pigs (Table 2).

### 3.2. Adipose Expression of Resistin and Visfatin

**Figure 1:**
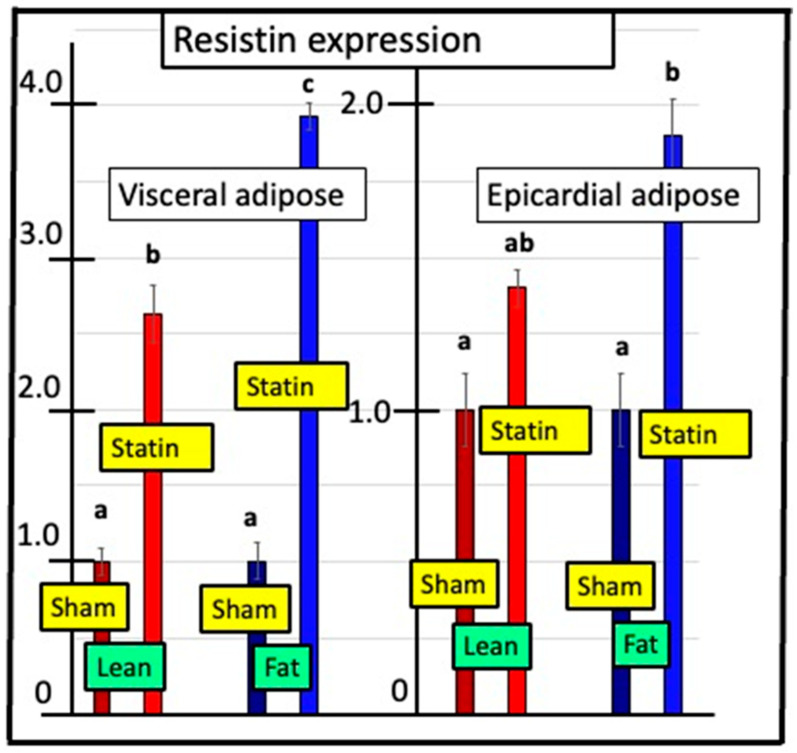
Effects of statin administration on resistin expression (RQ) in visceral and epicardial adipose tissue in lean and fat breeds of pigs (bars represents means ± SEM, ^a, b, c^ indicates difference at the level of *p* < 0.001, n = 3).

In both visceral and epicardial adipose tissue, expression of resistin was increased (*p* < 0.001) while that of visfatin was decreased (*p* < 0.001) in animals receiving statin administration compared to vehicle-treated controls (Table 3; Figure 1). There were both breed effects and interactions between statin treatment and breed on resistin expression (Table 3).

### 3.3. Plasma and Tissue Concentrations of Resistin

**Figure 2:**
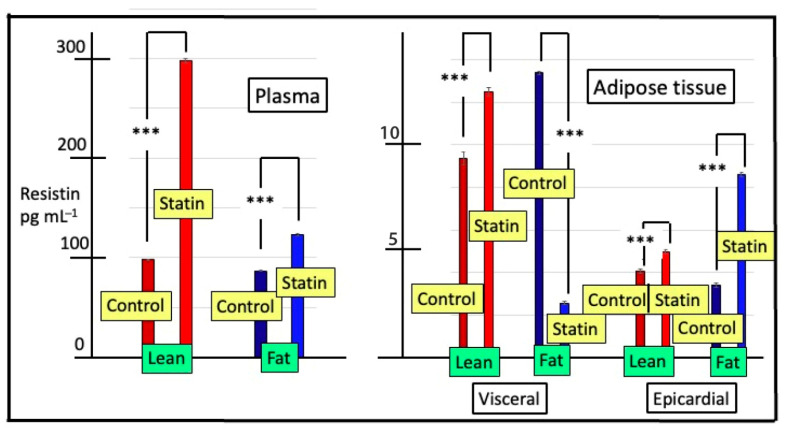
Effects of statin administration on concentrations of resistin in plasma (pg/mL) and visceral (ng/mg tissue) and epicardial (pg/mg tissue) adipose tissue in lean and fat breeds of pigs (bars represent means ± SEM, *** indicates difference *p* < 0.001, n = 5).

There were greater concentrations (*p* < 0.001) of resistin in the plasma, epicardial adipose tissue and pituitary gland in pigs receiving statin administration (Table 4, Figure 2).

In contrast, statin administration had effects of opposite directionality (*p* < 0.05) on concentrations of resistin in visceral adipose and hypothalamic tissues in the two breeds of pigs; this being reflected by the high interactions between statin treatment and breed (*p* < 0.001) (Table 4, Figure 2).

### 3.4. Plasma and Tissue Concentrations of Visfatin

There were opposite (*p* < 0.001) effects of statin treatment on concentrations of visfatin in plasma and epicardial adipose tissue in the two breeds (Table 5). Similarly, although concentrations of visfatin were elevated (*p* < 0.001) by statin treatment in visceral adipose tissue in the fat breed, there was no effect in lean breed pigs (Table 5). Concentrations of visfatin were decreased (*p* < 0.001) in hypothalamic and pituitary tissue from statin-treated pigs compared to sham controls (Table 4). Moreover, there were higher (*p* < 0.001) concentrations of visfatin in both hypothalamic and pituitary tissue from the fat, rather than lean, breed (Table 5).

### 3.5. Plasma Concentrations of Met-Enkephalin, Ghrelin, IL-6, TNFα, Leptin and Endothelin

Statin treatment exerted, or tended to exert, opposite directional effects on plasma concentrations of Met-enkephalin, ghrelin and IL-6 in pigs of the two breeds; this being seen from the marked interactions (*p* < 0.001) between effects of statin and breed (Table 6). In contrast, there were consistent effects of statin treatment on plasma concentrations of TNFα, leptin and endothelin. These were increased (*p* < 0.001) in pigs receiving statin treatment (Table 6). Moreover, there were higher (*p* < 0.001) plasma concentrations of both leptin and endothelin in the lean, rather than the fat, breed of pigs (Table 6).

### 3.6. Adipose Tissue Concentrations of Leptin, TNFα and Ghrelin

There were decreased (*p* < 0.001) concentrations of leptin in visceral adipose tissue from both lean and fat breeds of pigs receiving statin treatment (Table 7). In addition, visceral adipose concentrations of leptin were lower (*p* < 0.001) in the fat, rather than lean, breed (Table 7). Moreover, there were increased (*p* < 0.001) concentrations of ghrelin in epicardial adipose tissue from both lean and fat breeds of pigs receiving statin treatment (Table 7). Statin treatment had opposite direct effects on visceral adipose concentrations of TNFα being increased (*p* < 0.001) in lean breed pigs but decreased (*p* < 0.001) in tissue for fat breeds (Table 7). This was also reflected by the high (*p* < 0.001) interaction between effects of statin administration and breed (Table 7).

### 3.7. In Vitro Ghrelin Release from Hypothalamic, Pituitary and Adrenal Explants

Table 8 summarizes the effects of statin treatment and breed on in vitro ghrelin release from hypothalamic, pituitary and adrenal explants. Statin treatment in vivo enhanced (*p* < 0.001) basal release of ghrelin in vitro in hypothalamic explants from fat breed pigs, pituitary explants from lean breed pigs and adrenal tissue from both lean and fat breed pigs (Table 8).

## 4. Discussion

The present study examined whether metabolic stress induced by statin administration influenced adipocytes functioning based on synthesis, storage and release of adipokines and/or release of pro-inflammatory cytokines from macrophages and other immune cells present in adipose tissue. The simple answer is in the affirmative but with marked differences between responses in lean and fat breed pigs. It was hypothesized that there would not be breed differences in concentrations of stress hormones or adipokines between Pulawska and Polish Landrace in view of the report of no differences in either plasma concentration or adipose expression of a series of adipokines, including resistin and visfatin [19,20].

Effects of statin treatment can be assigned to four groups: 1. increased expression or concentration in both lean and fat pigs, 2. decreased expression or concentration in both lean and fat pigs, 3. increased or decreased in only one breed of pigs, and 4. statin treatment having opposite effects in both breeds but in opposite directions. Group 1 includes the expression of resistin in both visceral and epicardial adipose and plasma concentrations of resistin and leptin. Group 2 includes plasma concentrations of cortisol and endothelin together with expression of visfatin in both visceral and epicardial adipose tissue. Group 3 includes plasma concentrations of cholesterol, glucose and insulin. Group 4 includes visceral adipose tissue and hypothalamic concentrations of resistin, plasma concentrations of visfatin, Met-enkephalin, ghrelin and IL-6.

The basis for the difference is not immediately apparent but is presumed to be due to differences in metabolism and inflammatory responses.

There was hypercholesterolemia in the fat breed of pigs (Table 2). Plasma concentrations of cholesterol have been reported to be decreased in pigs by statin treatment particularly in pigs with diet-induced hypercholesterolemia [21,22]. Similarly, statin administration was accompanied by decreased plasma concentrations of cholesterol in the fat breed (Table 2). The effects of statin administration differed in the two breeds of pigs with no effect of statin in the lean breed (Table 2). Plasma concentrations of glucose were elevated in pigs with diet-induced hypercholesterolemia [22]; however, statin treatment had no effect on plasma concentrations of glucose in the pigs with hypercholesterolemia [22]. Similarly, statin treatment had no effect on plasma concentrations of glucose in fat pigs (Table 2). In contrast, plasma concentrations of glucose were lower in the lean breed pigs but increased with statin administration. In the present study, plasma concentrations of insulin were elevated in fat breed pigs receiving a statin administration despite there being no change in the plasma concentrations of glucose (Table 2). This may be interpreted as statin inducing insulin resistance. This may be similar to the reports of atorvastatin increasing glucose intolerance in type 2 diabetic mice [23] and statin use being associated with increased risk of diabetes [24].

HMG-CoA reductase is present in adrenocortical cells of another artiodactyl, cattle [25]. Plasma concentrations of the stress hormone, cortisol, were decreased in pigs of both breeds receiving statin treatment (Table 2). Similarly to the situation in the present in vivo study of pigs, in vitro cortisol production by bovine adrenocortical cells is depressed in the presence of a HMG-CoA reductase inhibitor [26,27]. In contrast, in a meta-analysis of human studies, plasma concentrations of cortisol were elevated by statin treatment [28].

Resistin can be considered as either an adipokine or a cytokine. Supporting the former, resistin is produced by adipocytes and pre-adipocytes [29,30] and, supporting the latter, resistin is produced by macrophages [30] and leukocytes [31]. Moreover, there is evidence that macrophages, rather than mature adipocytes, are the major source of resistin in human visceral adipose tissue [32] (reviewed: [33]).

There is limited information on plasma concentrations of resistin. Overall, plasma concentrations of resistin in the present study (Table 3, Figure 2) were similar to those reported in adult female mice [34] and young pigs [35] but were markedly higher than those reported in humans [36], horses [37] and sheep [38]. In the present study, adipose tissue expression and plasma concentrations of resistin were increased in pigs treated with statin (Table 3, Figure 1 and Figure 2). In contrast, atorvastatin decreased expression of resistin in human adipocytes in vitro [39]. Concentrations of resistin in visceral adipose tissue were decreased by statin treatment (Figure 2). Plasma concentrations of resistin have been reported to be elevated in horses with an inflammatory condition such as fever, tachycardia and leukocytosis–leukopenia [37]. However, in a meta-analysis of human studies, no effects of statin therapy were observed on plasma concentrations of resistin [40]. Some relationship between circulating concentrations of resistin and insulin resistance has been reported in humans with type 2 diabetes mellitus and obesity [41]. Statin therapy has been reported to be without effect on circulating concentrations of resistin [42,43].

There is limited information on the control of visfatin in pigs; however, visfatin expression in pigs has been characterized [44]. The major source of visfatin in human visceral adipose tissue is macrophages [32] (reviewed: [33]). In the present study, expression of visfatin was decreased in statin-treated pigs (Table 3), which was similar to the study that previously reported that statin treatment depressed expression/release of visfatin (reviewed: [45]).

There are changes in hypothalamic expression of visfatin during the estrous cycle of pigs [46]. Effects of visfatin on pituitary gene expression in pigs has been recently reported [47]. Plasma concentrations of visfatin were increased with statin, and it had a smaller effect in the fat breed (Table 3). In contrast, in rats, there were decreased circulating concentrations of visfatin with statin treatment [48]. Concentrations of visfatin in the epicardial, but not visceral, adipose were increased with statin administration in pigs (Table 3).

It was unexpected that the metabolic stress, statin, would influence pituitary concentrations of resistin and visfatin (Table 4). It is questioned whether pituitary contents of resistin and visfatin reflect production by adenohypophyseal cells or invading immune cells such as macrophages. The former is more likely as there is evidence that both resistin and visfatin are expressed in gonadotrophs [49]. There is also evidence for hypothalamic expression of visfatin in pigs with shifts during the estrous cycle [46].

There is cross-talk between ghrelin and pro-inflammatory responses. The ghrelin receptor (GHSR) is present in adipocytes with the binding of ghrelin increasing expression of fat storage related proteins [50]. Ghrelin is expressed in adipose tissue (reviewed [51]) and has also been reported in perivascular adipose tissue [52]. Ghrelin is also expressed in immune tissues (thymus, spleen and lymph nodes), neutrophils, B and T lymphocytes, and monocytes [53,54,55]. Ghrelin was not only present in both visceral and epicardial adipose tissue but also the concentrations were influenced by statin treatment (Table 5). The obtained results showed that both visceral and epicardial adipose tissue concentrations of ghrelin were increased in pigs receiving statin treatment (Table 7). Additionally, the in vitro basal ghrelin release from the hypothalamo–pituitary–adrenal axis of control fat pigs was significantly higher than in lean animals. Treatment with statin increased the basal ghrelin release from HPA axis of both breeds but to different extent (Table 8). Interestingly, naltrexone, an opioid receptors inhibitor added to tissue culture, modulated the ghrelin secretion from the hypothalamus, pituitary gland and adrenal gland (Table 8). It may be suggested that endogenous opioids, mainly Met-enkephalin, also regulate the ghrelin release and blood concentration in in vivo condition (Table 6). Similar results were shown in sheep stressed by isolation [16]. It is recognized that total ghrelin (i.e., both acylated and non-acylated) were determined in the present study.

It is suggested that pig epicardial adipose tissue may be a useful biomedical model. A role for epicardial adipose tissue in the development coronary atherosclerosis has been proposed with released factors acting on coronary arteries in a vasocrine manner (reviewed: [56,57]. Volumes of epicardial adipose tissue and circulating concentrations of adipokines/cytokines have been linked to coronary artery calcium concentrations [58].

## 5. Conclusions

**Scheme 1:**
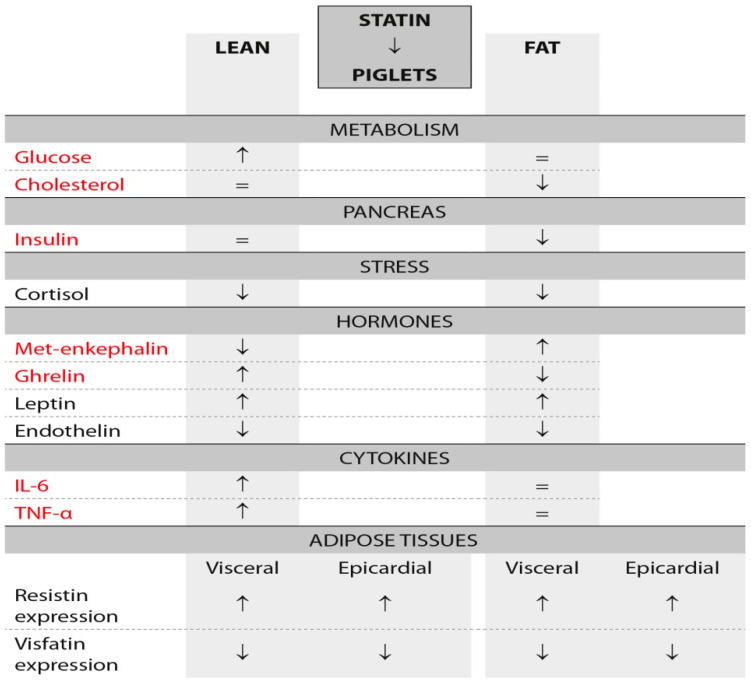
Differential responses of piglets (in red) to statin treatment.

Growing piglets may be a useful biomedical model for many physiological mechanisms during stress response such as metabolites changes, neurotransmitters and hormones activity. This model enables us to discover the differential responses dependent on breed/genetic background. It may be concluded that statins cause shifts in the expression and/or concentrations of both adipokines and inflammatory cytokines in adipose tissue (Scheme 1). However, future experiments are necessary to recognize the genes responsibility for differential response to statins treatment.

## Figures and Tables

**Table 1:** Primers employed.

Gene	F/R	Primers	bp (bp)	Accession No. Non-Number Number
18S rRNA	F R	5′-CTTTGGTCGCTCGCTCCTC-3′ 5′-CTGACCGGGTTGGTTTTGAT-3′	115	AY265350.1
Resistin Based on [14]	F R	5′-ATGAAGCCATCAATGAGA-3′ 5′-GCCTGAGGGGCAGGTGAC-3′	89	Nm_213783.1
Visfatin Based on [15]	F R	5′-CCAGTTGCTGATCCCAACAAA-3′ 5′-AAATTCCCTCCTGGTGTCCTATG-3′	95	XM_003132281.5

**Table 2:** Effect of statin treatment in vivo on plasma concentrations of cholesterol, glucose, insulin, and the stress hormone, cortisol, in lean (breed: PBZ) and fat (breed: Puławska) young pigs (means ± SEM, ^a, b, c, d^ *p* < 0.001, n = 5).

	Cholesterol mMol L^−1^	Glucose mMol L^−1^	Insulin pg mL^−1^	Cortisol ng mL^−1^
Lean breed (PBZ)				
Control	2.84 ± 0.11 ^a^	1.5 ± 0.09 ^a^	114 ± 4.31 ^a^	117 ± 3.12 ^d^
Statin	2.88 ± 0.09 ^a^	3.2 ± 0.16 ^b^	115 ± 3.44 ^a^	61.6 ± 1.36 ^b^
Fat breed (Puławska)				
Control	4.90 ± 0.10 ^b^	2.8 ± 0.51 ^b^	196 ± 4.00 ^b^	102 ± 2.60 ^c^
Statin	3.14 ± 0.09 ^a^	2.7 ± 0.43 ^b^	126 ± 3.71 ^a^	41.4 ± 0.93 ^a^

**Table 3:** Effect of statin treatment in vivo on the expression (relative quantification in real-time PCR, RQ) of resistin and visfatin in two adipose depots in lean (breed: PBZ) and fat (breed: Puławska) young pigs (means ±SEM ^a, b, c^ different letters indicate difference (*p* < 0.001, n = 5).

	Visceral Adipose		Epicardial Adipose	
	Resistin Expression	Visfatin Expression	Resistin Expression	Visfatin Expression
**Lean Breed (PBZ)**				
Control	1.00 ± 0.09 ^a^	1.00 ± 0.12 ^b^	1.00 ± 0.12 ^a^	1.00 ± 0.12 ^b^
Statin	2.63 ± 0.19 ^ab^	0.03 ± 0.006 ^a^	1.40 ± 0.06 ^a^	0.003 ± 0.001 ^a^
Fat breed (Puławska)				
Control	1.00 ± 0.12 ^a^	1.00 ± 0.09 ^b^	1.00 ± 0.12 ^a^	1.00 ± 0.12 ^b^
Statin	3.93 ± 0.09 ^c^	0.21 ± 0.018 ^a^	1.90 ± 0.12 ^b^	0.020 ± 0.0014 ^a^

**Table 4:** Effect of statin treatment in vivo on tissue concentrations of resistin (pg·mg^−1^) in hypothalamus and pituitary gland in lean (breed: PBZ) and fat (breed: Puławska) young pigs (means ± SEM, ^a, b, c, d^ *p* < 0.001, n = 5).

Groups	Tissue	
	Hypothalamus	Pituitary
Control lean	0.75 ± 0.09 ^b^	0.41 ± 0.04 ^b^
Statin lean	0.29 ± 0.02 ^c^	0.51 ± 0.03 ^c^
Control fat	0.22 ± 0.02 ^c^	1.81 ± 0.11 ^a^
Statin fat	0.19 ± 0.02 ^a^	1.62 ± 0.10 ^d^

**Table 5:** Effect of statin treatment in vivo on plasma and tissue concentrations of visfatin in two adipose depots in lean (breed: PBZ) and fat (breed: Puławska) young pigs (means ± SEM, n = 5, ^a, b, c, d^ *p* < 0.001, n = 5).

Breed and Treatment	Plasma Concentrations of Visfatin pg mL^−1^	Tissue Concentrations of Visfatin			
		Adipose Tissue		Hypothalamic pg mg^−1^	Pituitary Gland pg mg^−1^
		Visceral ng mg^−1^	Epicardial pg mg^−1^		
Lean breed					
Control	493 ± 10.7 ^d^	1.81 ± 0.017 ^a^	0.10 ± 0.007 ^a^	0.23 ± 0.012 ^b^	1.14 ± 0.029 ^b^
Statin	396 ± 8.7 ^c^	1.81 ± 0.016 ^a^	0.60 ± 0.015 ^b^	0.10 ± 0.011 ^a^	0.62 ± 0.015 ^a^
Fat breed					
Control	218 ± 1.03 ^a^	4.36 ± 0.093 ^c^	0.82 ± 0.017 ^c^	0.53 ± 0.011 ^c^	2.82 ± 0.019 ^d^
Statin	308 ± 1.28 ^b^	2.70 ± 0.071 ^b^	4.98 ± 0.030 ^d^	0.23 ± 0.009 ^b^	1.35 ± 0.021 ^c^

**Table 6:** Effect of statin treatment in vivo on plasma concentrations of Met-enkephalin, ghrelin, IL-6, TNFα, leptin and endothelin in lean (breed: PBZ) and fat (breed: Puławska) young pigs (means ± SEM, ^a, b, c, d^ *p* < 0.001, n = 5).

Breed/Treatment	Met-Enkephalin pg mL^−1^	Ghrelin ng mL^−1^	IL-6 pg mL^−1^	TNFα pg mL^−1^	Leptin ng mL^−1^	Endothelin pg mL^−1^
Lean						
Control	59.6 ± 2.03 ^b^	11.0 ± 0.71 ^a^	25.2 ± 0.86 ^b^	39.4 ± 0.93 ^b^	2.15 ± 0.019 ^a^	13.9 ± 0.09 ^c^
Statin	29.6 ± 0.87 ^d^	15.8 ± 1.16 ^ab^	30.2 ± 0.37 ^c^	41.8 ± 0.86 ^b^	2.53 ± 0.021 ^b^	10.0 ± 0.16 ^b^
Fat						
Control	42.4 ± 1.21 ^a^	17.6 ± 1.08 ^b^	15.4 ± 0.51 ^a^	33.4 ± 0.51 ^a^	2.72 ± 0.014 ^b^	10.4 ± 0.51 ^b^
Statin	64.0 ± 0.84 ^c^	13.6 ± 0.93 ^ab^	12.2 ± 1.06 ^a^	35.2 ± 0.66 ^a^	3.51 ± 0.038 ^c^	7.72 ± 0.43 ^a^

**Table 7:** Effect of statin treatment in vivo on adipose tissue concentrations of leptin, TNFα and ghrelin in lean (breed: PBZ) and fat (breed: Puławska) young pigs (means ± SEM, ^a, b, c, d^ *p* < 0.001, n = 5).

Breed/Treatment	Visceral Adipose Tissue		Epicardial Adipose Tissue	
	Leptin pg mg^−1^	TNFα ng mg^−1^	Leptin pg mg^−1^	Ghrelin pg mg^−1^
Lean				
Control	3.75 ± 0.030 ^a^	1.76 ± 0.019 ^a^	17.2 ± 0.196 ^a^	50.2 ± 1.36 ^a^
Statin	1.94 ± 0.026 ^a^	5.99 ± 0.054 ^c^	16.5 ± 0.201 ^a^	75.2 ± 1.07 ^c^
Fat				
Control	2.41 ± 0.023 ^c^	6.04 ± 0.025 ^c^	10.2 ± 0.093 ^c^	62.6 ± 1.03 ^b^
Statin	0.66 ± 0.017 ^b^	2.04 ± 0.020 ^b^	7.1 ± 0.086 ^b^	116 ± 3.03 ^d^

**Table 8:** In vitro ghrelin release as pg/mg tissue/30 min from hypothalamic, pituitary and adrenal explants from either control or statin-treated lean and fat breeds of pigs (means ± SEM, ^a, b, c^ *p* < 0.05, n = 5).

Breed/Treatment	Release of Ghrelin as pg/mg Tissue/30 min					
	Hypothalamic Tissue		Pituitary Tissue		Adrenal Tissue	
	Basal Release	Delta +Naltrexone	Basal Release	Delta +Naltrexone	Basal Release	Delta +Naltrexone
Lean						
Control	10.4 ± 0.51 ^a^	4.2 ± 0.58 ^a^	22.6 ± 0.51 ^a^	4.4 ± 0.81 ^a^	17.6 ± 0.51 ^a^	11.0 ± 1.38 ^b^
Statin	13.2 ± 0.37 ^ab^	2.8 ± 0.92 ^a^	31.0 ± 1.14 ^b^	6.0 ± 1.34 ^a^	39.4 ± 0.81 ^c^	10.0 ± 0.77 ^b^
Fat						
Control	14.8 ± 0.58 ^b^	10.0 ± 0.90 ^b^	32.0 ± 0.51 ^b^	2.8 ± 1.07 ^a^	29.4 ± 0.93 ^b^	4.4 ± 1.44 ^a^
Statin	23.2 ± 1.24 ^c^	10.0 ± 0.89 ^b^	31.8 ± 0.86 ^b^	12.0 ± 1.39 ^b^	41.2 ± 0.86 ^c^	9.0 ± 1.76 ^ab^

## Data Availability

The raw data supporting the conclusions of this article will be made available by the authors on request.

## Supplementary Materials

The following supporting information can be downloaded at: https://www.mdpi.com/article/10.3390/metabo15120797/s1, Table S1. Raw data of tested parameters. Table S2. Effect of statin treatment in vivo on plasma concentrations of cholesterol, glucose, insulin, and the stress hormone, cortisol, in lean (breed: PBZ) and fat (breed: Puławska) young pigs; Table S3. Effect of statin treatment in vivo on the expression of resistin and visfatin in two adipose depots in lean (breed: PBZ) and fat (breed: Puławska) young pigs; Table S4. Effect of statin treatment in vivo on tissue concentrations of resistin (pg mg^−1^) in hypothalamus and pituitary in lean (breed: PBZ) and fat (breed: Puławska) young pigs; Table S5. Effect of statin treatment in vivo on plasma and tissue concentrations of visfatin in two adipose depots in lean (breed: PBZ) and fat (breed: Puławska) young pigs; Table S6. Effect of statin treatment in vivo on plasma concentrations of Met-enkephalin, ghrelin, IL-6, TNFα, leptin and endothelin in lean (breed: PBZ) and fat (breed: Puławska) young pigs; Table S7. Effect of statin treatment in vivo on adipose tissue concentrations of leptin, TNFα, and ghrelin in lean (breed: PBZ) and fat (breed: Puławska) young pigs; Table S8. In vitro ghrelin release as pg/mg tissue/30 min from hypothalamic, pituitary and adrenal explants from either control or statin treated lean and fat breeds of pigs.
