# Supplementary material for: Metabolic Shifts Induced by Treatment with Statin Influences Circulating Concentrations of the Stress Hormone, Cortisol, but Has Different Effects on Selected Cytokines, Adipokines and Neuropeptides in Lean and Fat Lines of Young Pigs
Source: Metabolites. 2025 Dec 16;15(12):797. doi: 10.3390/metabo15120797 (PMC12735346; doi:10.3390/metabo15120797)
Supplement: Supplementary file 1 [file metabolites-15-00797-s001.zip › metabolites-3991209-supplementary.pdf]

Table S1. Raw data of tested parameters.

Piglets , 10-12 kg, female, two strains: 1.Puławska -wild without genetic interbreeding, slowly growing, a lot of fat; 2. PBZ -Polish White, many breeds, growing fast, small amount of fat, N=5/group, Groups: Control ;statin for 5 days, (HMG-CoA reductase inhibitor)

IL- levels in piglets pg/ml n=5,

Strain/group	control	statin
PBZ	25<br>24<br>23<br>28<br>26	30<br>28<br>26<br>33<br>34
Puławska	16<br>14<br>17<br>15<br>15	12<br>9<br>11<br>14<br>15

Cortisol ng/ml

Strain/group	control	statin
PBZ	118<br>112<br>122<br>109<br>126	62<br>59<br>58<br>65<br>64
Puławska	101<br>99<br>112<br>102<br>97	43<br>41<br>40<br>39<br>44

Group/strain	PBZ resistin pg/mg hypothalamus	Puławska resistin pg/mg hypothalamus	PBZ visfatin pg/mg hypothalamus	Puławska visfatin pg/mg hypothalamus
Control	0.75<br>0.81<br>0.71<br>0.72<br>0.73	0.22<br>0.25<br>0.21<br>0.19<br>0.24	0.22<br>0.25<br>0.23<br>0.25<br>0.19	0.53<br>0.59<br>0.49<br>0.50<br>0.53
Statin	0.29<br>0.33<br>0.31<br>0.27<br>-	0.19<br>0.23<br>0.22<br>0.18<br>0.17	0.10<br>0.11<br>0.12<br>0.08<br>0.08	0.23<br>0.25<br>0.22<br>0.21<br>0.26
	pituitary	pituitary	pituitary	pituitary
Control	0.41<br>0.43<br>0.42<br>0.39<br>0.44	1.81<br>1.75<br>1.81<br>1.84<br>1.83	1.15<br>1.22<br>1.11<br>1.05<br>1.18	2.83<br>2.88<br>2.84<br>2.77<br>2.79
Statin	0..51<br>0.55<br>0.52<br>0.48<br>0.49	1.62<br>1.66<br>1.59<br>1.63<br>1.60	0.62<br>0.66<br>0.58<br>0.59<br>0.64	1.35<br>1.41<br>1.37<br>1.29<br>1.32

White adipose tissue -visceral

Group/strain	PBZ resistin ng/mg	Puławska Resistin ng/mg	PBZ visfatin Pg/mg	Puławska Visfatin pg/mg	PBZ Leptin Ng/g	Puławska Leptin ng/g	PBZ TNFalfa Pg/g	Puławska TNFalfa Pg/g
Control	9.1<br>10.2<br>9.9<br>8.7<br>8.9	13.5<br>12.9<br>13.9<br>13.1<br>13.4	1.80<br>1.76<br>1.79<br>1.86<br>1.82	4.4<br>4.2<br>4.1<br>4.5<br>4.6	3.75<br>3.66<br>3.82<br>3.72<br>3.81	2.41<br>2.38<br>2.35<br>2.44<br>2.48	1750<br>1810<br>1790<br>1710<br>1721	6050<br>6090<br>6100<br>5990<br>5980
Statin	12.5<br>12.2<br>12.8<br>11.9<br>12.9	2.5<br>2.4<br>2.2<br>2.8<br>2.9	1.82<br>1.84<br>1.78<br>1.85<br>1.77	2.7<br>2.8<br>2.9<br>2.5<br>2.6	1.90<br>1.99<br>2.01<br>1.88<br>1.91	0.66<br>0.71<br>0.67<br>0.61<br>0.64	5990<br>6120<br>6060<br>5800<br>5970	2050<br>2100<br>2090<br>1990<br>1980

Table S2. Effect of statin treatment *in vivo* on plasma concentrations of cholesterol, glucose, insulin, and the stress hormone, cortisol, in lean (breed: PBZ) and fat (breed: Puławska) young pigs (X±SEM, $^{a, b, c, d}$ P < 0.05, n=5)

	Cholesterol mMol L^{-1}	Glucose mMol L^{-1}	Insulin pg mL^{-1}	Cortisol ng mL^{-1}
Lean breed (PBZ)				
Control	2.84 ± 0.11^a	1.5 ± 0.09^a	114 ± 4.31^a	117 ± 3.12^d
Statin	2.88 ± 0.09^a	3.2 ± 0.16^b	115 ± 3.44^a	61.6 ± 1.36^b
Fat breed (Puławska)				
Control	4.90 ± 0.10^b	2.8 ± 0.51^b	196 ± 4.00^b	102 ± 2.60^c
Statin	3.14 ± 0.09^a	2.7 ± 0.43^b	126 ± 3.71^a	41.4 ± 0.93^a
2-way ANOVA				
Statin	F = 78.7 (P = 1.42E^{-7})	F = 54.2 (P = 1.16E^{-6})	F = 78.7 (P = 1.42E^{-7})	F = 707 (P = 1.14E^{-14})
Breed	F = 143 (P = 2.15E^{-9})	F = 12.6 (P = 0.0027)	F = 2.15 (P = 1.42E^{-7})	F = 65.2 (P = 4.91E^{-7})
Interaction	F = 86.2 (P = 7.66E^{-8})	F = 68.4 (P = 3.58E^{-7})	F = 86.2 (P = 7.66E^{-8})	F = 1.3 (NS)

$^{a, b, c, d}$ Different letters indicate difference (P < 0.05)
NS not significant

Table S3. Effect of statin treatment *in vivo* on the expression of resistin and visfatin in two adipose depots in lean (breed: PBZ) and fat (breed: Puławska) young pigs (X±SEM, ^{a,b,c} P<0.05, n=5)

	Visceral adipose		Epicardial adipose	
	Resistin expression	Visfatin expression	Resistin expression	Visfatin expression
Lean breed (PBZ)				
Control	1.00 ± 0.09^a	1.00 ± 0.12^b	1.00 ± 0.12^a	1.00 ± 0.12^b
Statin	2.63 ± 0.19^{ab}	0.03 ± 0.006^a	1.40 ± 0.06^a	0.003 ± 0.001^a
Fat breed (Puławska)				
Control	1.00 ± 0.12^a	1.00 ± 0.09^b	1.00 ± 0.12^a	1.00 ± 0.12^b
Statin	3.93 ± 0.09^c	0.21 ± 0.018^a	1.90 ± 0.12^b	0.020 ± 0.0014^a
2-way ANOVA				
Statin	F = 324 (P = 9.25E^{-8})	F = 115 (P = 5.01E^{-6})	F = 39 (P = 0.0002)	F = 147 (P = 2.01E^{-6})
Breed	F = 25.3 (P = 0.001)	F = 1.2 NS	F = 5.77 (P = 0.043)	F = 0.01 NS
Interaction	F = 28.1 (P = 0.0007)	F = 1.2 NS	F = 5.77 (P = 0.043)	F = 0.01 NS

$^{a, b, c}$ Different letters indicate difference (P < 0.05)
NS not significant

Table S4. Effect of statin treatment *in vivo* on tissue concentrations of resistin (pg mg^{-1}) in hypothalamus and pituitary in lean (breed: PBZ) and fat (breed: Puławska) young pigs (means±SEM, ^{a,b,c,d} P<0.001, n=5).

Breed and treatment	Plasma concentrations of resistin pg mL^{-1}	Tissue concentrations of resistin			
		Adipose tissue		Hypothalamic pg mg^{-1}	Pituitary gland pg mg^{-1}
		Visceral ng mg^{-1}	Epicardial pg mg^{-1}		
Lean breed					
Control	98 ± 0.63^{b}	9.36 ± 0.29^{b}	4.06 ± 0.09^{b}	0.75 ± 0.09^{b}	0.41 ± 0.04^{b}
Statin	298 ± 2.41^{d}	12.5 ± 0.19^{c}	4.98 ± 0.10^{c}	0.29 ± 0.02^{c}	0.51 ± 0.03^{c}
Fat breed					
Control	87 ± 1.03^{a}	13.4 ± 0.07^{c}	3.42 ± 0.10^{a}	0.22 ± 0.02^{c}	1.81 ± 0.11^{a}
Statin	123 ± 1.28^{c}	2.56 ± 0.06^{a}	8.62 ± 0.11^{d}	0.19 ± 0.02^{a}	1.62 ± 0.10^{d}
2-way ANOVA					
Statin	F = 707 (*P* = 1.14E^{-14})	F = 356 (*P* = 2.33E^{-12})	F = 226 (*P* = 7.36E^{-11})	F = 356 (*P* = 2.33E^{-12})	F = 226 (*P* = 7.36E^{-11})
Breed	F = 65.2 (*P* = 4.91E^{-7})	F = 209 (*P* = 1.32E^{-10})	F = 941 (*P* = 3.24E^{-15})	F = 209 (*P* = 1.32E^{-10})	F = 941 (*P* = 3.24E^{-15})
Interaction	F = 1.3 (NS)	F = 1161 (*P* = 2.03E^{-16})	F = 460 (*P* = 3.24E^{-13})	F = 1161 (*P* = 2.03E^{-16})	F = 460 (*P* = 3.24E^{-13})

$^{a, b, c, d}$ Different letters indicate difference (*P* < 0.05)
NS not significant

Table S5. Effect of statin treatment *in vivo* on plasma and tissue concentrations of visfatin in two adipose depots in lean (breed: PBZ) and fat (breed: Puławska) young pigs (X±SEM, ^{a,b,c,d}P<0.05, n=5)

Breed and treatment	Plasma concentrations of visfatin pg mL^{-1}	Tissue concentrations of visfatin			
		Adipose tissue		Hypothalamic pg mg^{-1}	Pituitary gland pg mg^{-1}
		Visceral ng mg^{-1}	Epicardial pg mg^{-1}		
Lean breed					
Control	493 ± 10.7^{d}	1.81 ± 0.017^{a}	0.10 ± 0.007^{a}	0.23 ± 0.012^{b}	1.14 ± 0.029^{b}
Statin	396 ± 8.7^{c}	1.81 ± 0.016^{a}	0.60 ± 0.015^{b}	0.10 ± 0.011^{a}	0.62 ± 0.015^{a}
Fat breed					
Control	218 ± 1.03^{a}	4.36 ± 0.093^{c}	0.82 ± 0.017^{c}	0.53 ± 0.011^{c}	2.82 ± 0.019^{d}
Statin	308 ± 1.28^{b}	2.70 ± 0.071^{b}	4.98 ± 0.030^{d}	0.23 ± 0.009^{b}	1.35 ± 0.021^{c}
2 way ANOVA					
Statin	F = 0.07 NS	F = 196 (*P* = 2.34E^{-10})	F = 14,589 (*P* = 4.07E^{-25})	F = 311 (*P* = 4.32E^{-12})	F = 2127 (*P* = 1.90E^{-18})
Breed	F = 257 (*P* = 4.91E^{-7})	F = 838 (*P* = 2. 99E^{15})	F = 17,531 (*P* = 9.39E^{-26})	F = 329 (*P* = 6.59E^{-12})	F = 3096 (*P* = 9.61E^{-20})
Interaction	F = 68.1 (*P* = 3.69E^{-7})	F = 196 (*P* = 2.11E^{-10})	F = 8928 (*P* = 2.06E^{-23})	F = 46.5 (*P* = 4.11E^{-6})	F = 481 (*P* = 2.30E^{-13})

$^{a, b, c}$ Different letters indicate difference (*P* < 0.05)
NS not significant

Table S6. Effect of statin treatment *in vivo* on plasma concentrations of Met-enkephalin, ghrelin, IL-6, TNFα, leptin and endothelin in lean (breed: PBZ) and fat (breed: Puławska) young pigs (X±SEM, ^{a,b,c,d} P<0.05, n=5)

Breed/Treatment	Met-enkephalin pg mL^{-1}	Ghrelin ng mL^{-1}	IL-6 pg mL^{-1}	TNFα pg mL^{-1}	Leptin ng mL^{-1}	Endothelin pg mL^{-1}
Lean						
Control	59.6 ± 2.03^{b}	11.0 ± 0.71^{a}	25.2 ± 0.86^{b}	39.4 ± 0.93^{b}	2.15 ± 0.019^{a}	13.9 ± 0.09^{c}
Statin	29.6 ± 0.87^{d}	15.8 ± 1.16^{ab}	30.2 ± 0.37^{c}	41.8 ± 0.86^{b}	2.53 ± 0.021^{b}	10.0 ± 0.16^{b}
Fat						
Control	42.4 ± 1.21^{a}	17.6 ± 1.08^{b}	15.4 ± 0.51^{a}	33.4 ± 0.51^{a}	2.72 ± 0.014^{b}	10.4 ± 0.51^{b}
Statin	64.0 ± 0.84^{c}	13.6 ± 0.93^{ab}	12.2 ± 1.06^{a}	35.2 ± 0.66^{a}	3.51 ± 0.038^{c}	7.72 ± 0.43^{a}
2-way ANOVA						
Statin	F = 9.97 (*P* = 0.006)	F = 0.17 NS	F = 0.74 NS	F = 7.7 (*P* = 0.014)	F = 544 (*P* = 8.82E^{-14})	F = 92.4 (*P* = 4.76E^{-8})
Breed	F = 41.8 (*P* = 7.82E^{-6})	F = 5.01 (*P* = 0.040)	F = 176 (*P* = 4.66E^{-10})	F = 69.0 (*P* = 3.38E^{-7})	F = 962 (*P* = 1.01E^{-15})	F = 70.5 (*P* = 2.95E^{-7})
Inter-action	F = 376 (*P* = 1.54E^{-12})	F = 20.1 (*P* = 0.0004)	F = 15.4 (*P* = 0.001)	F = 0.7 NS	F = 69.5 (*P* = 3.24E^{-7})	F = 2.05 NS

$^{a, b, c}$ Different letters indicate difference ($P < 0.05$)
NS not significant

Table S7. Effect of statin treatment *in vivo* on adipose tissue concentrations of leptin, TNFα, and ghrelin in lean (breed: PBZ) and fat (breed: Puławska) young pigs (X±SEM, ^{a,b,c,d}P<0.001, n=5)

Breed/Treatment	Visceral adipose tissue		Epicardial adipose tissue	
	Leptin pg mg^{-1}	TNFα ng mg^{-1}	Leptin pg mg^{-1}	Ghrelin pg mg^{-1}
Lean				
Control	3.75 ± 0.030^{a}	1.76 ± 0.019^{a}	17.2 ± 0.196^{a}	50.2 ± 1.36^{a}
Statin	1.94 ± 0.026^{a}	5.99 ± 0.054^{c}	16.5 ± 0.201^{a}	75.2 ± 1.07^{c}
Fat				
Control	2.41 ± 0.023^{c}	6.04 ± 0.025^{c}	10.2 ± 0.093^{c}	62.6 ± 1.03^{b}
Statin	0.66 ± 0.017^{b}	2.04 ± 0.020^{b}	7.1 ± 0.086^{b}	116 ± 3.03^{d}
2-way ANOVA				
Statin	F = 5450 (*P* = 1.06E^{-21})	F = 11.9 (*P* = 0.003)	F = 156 (*P* = 1.15E^{-9})	F = 464 (*P* = 3.03E^{-13})
Breed	F = 2939 (*P* = 1.46E^{-19})	F = 2.76 (*P* = 0.01)	F = 2855 (*P* = 1.82E^{-19})	F = 214 (*P* = 1.12E^{-10})
Interaction	F = 1.54	F = 15,010	F = 61.2	F = 60.9

	NS	(P = 3.24E^{-25})	(P = 7.45E^{-7})	(P = 7.62E^{-7})

[a, b, c] Different letters indicate difference ($P < 0.001$)

NS not significant

TABLE S8. *In vitro* ghrelin release as pg/mg tissue/30 min from hypothalamic, pituitary and adrenal explants from either control or statin treated lean and fat breeds of pigs (X±SEM, [a,b,c]P<0.001, n=5)

Breed/Treatment	Release of ghrelin as pg/mg tissue/30 min					
	Hypothalamic tissue		Pituitary tissue		Adrenal tissue	
	Basal release	Delta<br>+ Naltrexone	Basal release	Delta<br>+ Naltrexone	Basal release	Delta<br>+ Naltrexone
Lean						
Control	10.4 ± 0.51[a]	4.2 ± 0.58[a]	22.6 ± 0.51[a]	4.4 ± 0.81[a]	17.6 ± 0.51[a]	11.0 ± 1.38[b]
Statin	13.2 ± 0.37[ab]	2.8 ± 0.92[a]	31.0 ± 1.14[b]	6.0 ± 1.34[a]	39.4 ± 0.81[c]	10.0 ± 0.77[b]
Fat						
Control	14.8 ± 0.58[b]	10.0 ± 0.90[b]	32.0 ± 0.51[b]	2.8 ± 1.07[a]	29.4 ± 0.93[b]	4.4 ± 1.44[a]
Statin	23.2 ± 1.24[c]	10.0 ± 0.89[b]	31.8 ± 0.86[b]	12.0 ± 1.39[b]	41.2 ± 0.86[c]	9.0 ± 1.76[ab]
2-way ANOVA						
Statin	F = 90.9<br>(P = 1.45E^{-6})	F = 0.7<br>(*NS*)	F = 24.0<br>(P < 0.0002)	F = 20.4<br>(P = 0.0003)	F = 448<br>(P = 3.99E^{-13})	F = 1.68<br>(NS)
Breed	F = 55.0<br>(P = 5.29E^{-8})	F = 58.7<br>(P = 9.96E^{-7})	F = 37.1<br>(P = 1.55E^{-5})	F = 3.40<br>(NS)	F = 73.4<br>(P = 2.26E^{-7})	F = 7.54<br>(P = 0.014)
Interaction	F = 13.8<br>(P = 0.002)	F = 0.7<br>(NS)	F = 26.4<br>(P = 9.89E^{-5})	F = 10.1<br>(P = 0.006)	F = 39.7<br>(P = 1.06E^{-5})	F = 4.09<br>(NS)

[a, b, c] Different letters indicate difference ($P < 0.05$)

NS not significant
